# 5,6-Di­oxo-1,10-phenanthrolin-1-ium trifluoro­methane­sulfonate

**DOI:** 10.1107/S1600536808016632

**Published:** 2008-07-19

**Authors:** Jonathan Onuegbu, Ray J. Butcher, Charles Hosten, Uche Charles Udeochu, Oladapo Bakare

**Affiliations:** a Department of Chemistry, Howard University, 525 College Street NW, Washington, DC 20059, USA

## Abstract

In the structure of the title salt, C_12_H_7_N_2_O_2_^+^·CF_3_SO_3_^−^, the cation participates in hydrogen bonding with the dione group of an adjacent cation as well as with the trifluoro­methane­sulfonate anion. In addition, there is an extensive network of C—H⋯O inter­actions between the cations and anions. There are two formula units per asymmetric unit. The crystal studied exhibits inversion twinning.

## Related literature

For literature on the coordinating ability of phendione, see: Calderazzo *et al.* (1999 ▶, 2002 ▶); Calucci *et al.* (2006 ▶); Fox *et al.* (1991 ▶); Galet *et al.* (2005 ▶); Lei *et al.* (1996 ▶); Okamura *et al.* (2006 ▶); Paw & Eisenberg (1997 ▶); Ruiz *et al.* (1999 ▶); Shavaleev *et al.* (2003*a* ▶,*b* ▶); Ma *et al.* (2002 ▶). For our own reports on phendione, see: Onuegbu *et al.* (2007 ▶); Udeochu *et al.* (2007 ▶).
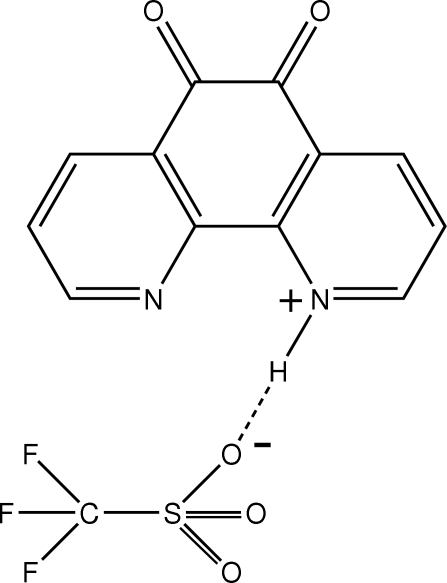


## Experimental

### 

#### Crystal data

C_12_H_7_N_2_O_2_^+^·CF_3_O_3_S^−^
*M* *_r_* = 360.27
Monoclinic, 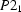

*a* = 6.4896 (2) Å
*b* = 16.3963 (5) Å
*c* = 13.2430 (3) Å
β = 94.393 (2)°
*V* = 1404.99 (7) Å^3^
*Z* = 4
Mo *K*α radiation
μ = 0.30 mm^−1^
*T* = 200 (2) K
0.51 × 0.22 × 0.18 mm

#### Data collection

Oxford Diffraction Gemini R diffractometer
Absorption correction: multi-scan (*SCALE3 ABSPACK*; Oxford Diffraction, 2007 ▶) *T*_min_ = 0.897, *T*_max_ = 1.000 (expected range = 0.850–0.948)
13319 measured reflections
7960 independent reflections
5208 reflections with *I* > 2σ(*I*)
*R*_int_ = 0.023

#### Refinement

*R*[*F* ^2^ > 2σ(*F* ^2^)] = 0.034
*wR*(*F* ^2^) = 0.076
*S* = 0.94
7960 reflections
434 parameters
1 restraint
H-atom parameters constrained
Δρ_max_ = 0.24 e Å^−3^
Δρ_min_ = −0.38 e Å^−3^
Absolute structure: Flack (1983 ▶), with 2713 Friedel pairs
Flack parameter: 0.40 (5)

### 

Data collection: *CrysAlis CCD* (Oxford Diffraction, 2007 ▶); cell refinement: *CrysAlis RED* (Oxford Diffraction, 2007 ▶); data reduction: *CrysAlis RED*; program(s) used to solve structure: *SHELXS97* (Sheldrick, 2008 ▶); program(s) used to refine structure: *SHELXL97* (Sheldrick, 2008 ▶); molecular graphics: *SHELXTL* (Sheldrick, 2008 ▶); software used to prepare material for publication: *SHELXTL*.

## Supplementary Material

Crystal structure: contains datablocks global, I. DOI: 10.1107/S1600536808016632/ng2417sup1.cif

Structure factors: contains datablocks I. DOI: 10.1107/S1600536808016632/ng2417Isup2.hkl

Additional supplementary materials:  crystallographic information; 3D view; checkCIF report

## Figures and Tables

**Table 1:** Hydrogen-bond geometry (Å, °)

*D*—H⋯*A*	*D*—H	H⋯*A*	*D*⋯*A*	*D*—H⋯*A*
N1*A*—H1*AB*⋯O22	0.88	2.01	2.830 (2)	154
N1*B*—H1*BB*⋯O12	0.88	2.02	2.835 (2)	154

**Fig. 1.**
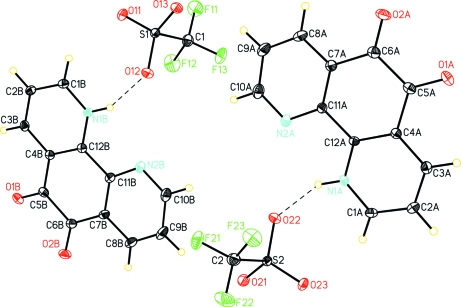
View of the two formula units in the asymmetric unit showing the atom-labeling scheme. Dotted lines indicate the hydrogen bonding interactions. Displacement ellipsoids are drawn at the 20% probability level.

**Fig. 2.**
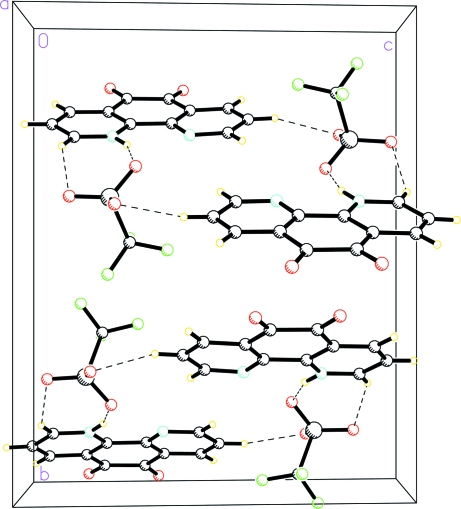
The molecular packing of (I) viewed approximately along the b axis. Dotted lines indicate the hydrogen bonding interactions.

## supplementary crystallographic information

### Comment

Phendione (1,10-phenanthroline-5,6-dione) is an excellent ligand that incorporates two functional groups with different coordination properties (Ma *et al.*, 2002; Calderazzo *et al.*, 1999, 2002; Calucci *et al.*, 2006; Galet *et al.*, 2005; Lei *et al.*, 1996; Okamura *et al.*, 2006). This well known ligand posssesses both the α-diimine and orthoquinone moieties. While phendione usually binds to metals through its imine N atoms, in some cases both the N and O donors are used simultaneously (Calderazzo *et al.*, 1999; Fox *et al.*, 1991; Shavaleev *et al.*, 2003*a*,*b*; Ruiz *et al.*, 1999; Paw & Eisenberg, 1997). In this paper as part of our study of phendione chemistry (Udeochu *et al.*, 2007; Onuegbu *et al.*, 2007) we report the synthesis and characterization of the trifluoromethanesulfonate salt of mono-protonated 1,10-phenanthroline-5,6-dione.

The structure consists of a mono-protonated phendione cation and a trifluoromethanesulfonate (CF_3_SO_3_^-^) anion. The C═O bond lengths in the phendione cation (1.208 (2), 1.209 (2) and the metrical parameters involving the phendione N atoms are comparable in value to those found in neutral 1,10-phenanthroline-5,6-dione.

The N—H protons participate in hydrogen bonds with adjoining phendione cations. In addition there is an extensive network of weak C—H···O interactions to both phendione O and trifluoromethanesulfonate O atoms.

### Experimental

A flask containing 1,10-phenanthroline hydrate (1.00 g, 5.04 mmol) and potassium bromide (5.95 g, 50.0 mmol) was placed in an ice bath. Concentrated sulfuric acid (20 ml) was added in small portions, followed by drop wise addition of concentrated nitric acid (10 ml). The resulting solution was heated for 2 h at 80–85° C and cooled to room temperature. The solution was then poured into 400 ml water and neutralized with sodium bicarbonate, after which the phendione was extracted with dichloromethane, and recrystallized using a methanol–water mixture.

The title compound was synthesized in an atmosphere saturated with N_2_. To a solution of silver trifluoromethanesulfonate (0.079 g) in 10 ml CH_3_CN (acidified to pH 2 using concentrated triflic acid), was added a solution (10 ml) of CH_3_CN containing 0.065 g of phendione (acidified to pH 2 using triflic acid). The final yellowish solution was filtered and allowed to slowly evaporate yield reddish brown crystals of the title compound.

### Refinement

H atoms were placed in geometrically idealized positions and constrained to ride on their parent atoms with a C—H distance of 0.95 Å and *U*_iso_(H) = 1.2*U*_eq_(C). The H atoms attached to N in the phendione cation were idealized with an N—H distance of 0.88 Å.

### Crystal data

**Table:**

C_12_H_7_N_2_O_2_^+^·CF_3_O_3_S^–^	*F*_000_ = 728
*M**_r_* = 360.27	*D*_x_ = 1.703 Mg m^−^^3^
Monoclinic, *P*2_1_	Mo *K*α radiation λ = 0.71073 Å
*a* = 6.4896 (2) Å	Cell parameters from 5857 reflections
*b* = 16.3963 (5) Å	θ = 4.7–32.5º
*c* = 13.2430 (3) Å	µ = 0.30 mm^−^^1^
β = 94.393 (2)º	*T* = 200 (2) K
*V* = 1404.99 (7) Å^3^	Needle, yellow-orange
*Z* = 4	0.51 × 0.22 × 0.18 mm

### Data collection

**Table:**

Oxford Diffraction Gemini diffractometer	7960 independent reflections
Radiation source: fine-focus sealed tube	5208 reflections with *I* > 2σ(*I*)
Monochromator: graphite	*R*_int_ = 0.023
Detector resolution: 10.5081 pixels mm^-1^	θ_max_ = 32.5º
*T* = 200(2) K	θ_min_ = 4.7º
φ and ω scans	*h* = −9→7
Absorption correction: multi-scan[Empirical absorption correction using spherical harmonics, implemented in SCALE3 ABSPACK scaling algorithm (Oxford Diffraction, 2007)]	*k* = −24→21
*T*_min_ = 0.897, *T*_max_ = 1.000	*l* = −19→19
13319 measured reflections	

### Refinement

**Table:**

Refinement on *F*^2^	Hydrogen site location: inferred from neighbouring sites
Least-squares matrix: full	H-atom parameters constrained
*R*[*F*^2^ > 2σ(*F*^2^)] = 0.034	*w* = 1/[σ^2^(*F*_o_^2^) + (0.0394*P*)^2^] where *P* = (*F*_o_^2^ + 2*F*_c_^2^)/3
*wR*(*F*^2^) = 0.077	(Δ/σ)_max_ = 0.001
*S* = 0.94	Δρ_max_ = 0.24 e Å^−^^3^
7960 reflections	Δρ_min_ = −0.38 e Å^−^^3^
434 parameters	Extinction correction: none
1 restraint	Absolute structure: Flack (1983), with 2713 Friedel pairs
Primary atom site location: structure-invariant direct methods	Flack parameter: 0.40 (5)
Secondary atom site location: difference Fourier map	

### Special details

**Table:**

Experimental. The data were measured to a 2θ limit of 50 °, but the low completeness was caused by
Geometry. All e.s.d.'s (except the e.s.d. in the dihedral angle between two l.s. planes) are estimated using the full covariance matrix. The cell e.s.d.'s are taken into account individually in the estimation of e.s.d.'s in distances, angles and torsion angles; correlations between e.s.d.'s in cell parameters are only used when they are defined by crystal symmetry. An approximate (isotropic) treatment of cell e.s.d.'s is used for estimating e.s.d.'s involving l.s. planes.
Refinement. Refinement of *F*^2^ against ALL reflections. The weighted *R*-factor *wR* and goodness of fit *S* are based on *F*^2^, conventional *R*-factors *R* are based on *F*, with *F* set to zero for negative *F*^2^. The threshold expression of *F*^2^ > σ(*F*^2^) is used only for calculating *R*-factors(gt) *etc*. and is not relevant to the choice of reflections for refinement. *R*-factors based on *F*^2^ are statistically about twice as large as those based on *F*, and *R*- factors based on ALL data will be even larger.

### Fractional atomic coordinates and isotropic or equivalent isotropic displacement parameters (Å^2^)

**Table:**

	*x*	*y*	*z*	*U*_iso_*/*U*_eq_	
S1	0.64118 (7)	0.25922 (3)	0.84492 (4)	0.02642 (11)	
S2	0.86022 (7)	0.38484 (3)	0.23321 (3)	0.02618 (11)	
F11	0.7237 (3)	0.10424 (9)	0.86156 (13)	0.0732 (5)	
F12	0.9730 (2)	0.17770 (11)	0.81474 (14)	0.0735 (5)	
F13	0.7153 (2)	0.14825 (10)	0.70995 (11)	0.0604 (4)	
F21	0.7945 (2)	0.49049 (10)	0.37563 (11)	0.0648 (4)	
F22	0.7727 (3)	0.54033 (10)	0.22511 (15)	0.0759 (5)	
F23	0.5298 (2)	0.46511 (10)	0.27365 (13)	0.0636 (4)	
O11	0.7135 (2)	0.26580 (11)	0.94979 (10)	0.0419 (4)	
O12	0.7202 (2)	0.32177 (10)	0.78073 (11)	0.0340 (3)	
O13	0.4246 (2)	0.24330 (10)	0.82584 (11)	0.0376 (4)	
O21	1.0772 (2)	0.40075 (10)	0.25085 (11)	0.0390 (4)	
O22	0.7848 (2)	0.32033 (9)	0.29570 (11)	0.0346 (4)	
O23	0.7822 (2)	0.38146 (11)	0.12885 (10)	0.0422 (4)	
O1A	−0.2484 (2)	0.12049 (10)	0.20079 (13)	0.0400 (4)	
O2A	−0.2548 (2)	0.12362 (10)	0.40583 (13)	0.0457 (4)	
O1B	1.7556 (2)	0.51876 (9)	0.87690 (12)	0.0386 (4)	
O2B	1.7531 (2)	0.52437 (10)	0.67146 (12)	0.0431 (4)	
N1A	0.4031 (2)	0.24850 (11)	0.22669 (12)	0.0254 (4)	
H1AB	0.5045	0.2692	0.2666	0.030*	
N2A	0.4147 (3)	0.24527 (12)	0.42945 (13)	0.0319 (4)	
N1B	1.1004 (2)	0.39478 (11)	0.85110 (12)	0.0258 (4)	
H1BB	1.0012	0.3723	0.8115	0.031*	
N2B	1.0923 (3)	0.39406 (12)	0.64828 (13)	0.0327 (4)	
C1	0.7697 (4)	0.16729 (15)	0.80584 (17)	0.0387 (6)	
C2	0.7337 (4)	0.47486 (15)	0.27958 (18)	0.0402 (6)	
C1A	0.4191 (3)	0.25021 (14)	0.12634 (15)	0.0314 (5)	
H1AA	0.5384	0.2729	0.0999	0.038*	
C2A	0.2629 (3)	0.21907 (14)	0.06179 (17)	0.0322 (5)	
H2AA	0.2737	0.2197	−0.0093	0.039*	
C3A	0.0900 (3)	0.18683 (13)	0.10145 (17)	0.0301 (5)	
H3AA	−0.0202	0.1658	0.0577	0.036*	
C4A	0.0781 (3)	0.18525 (12)	0.20630 (15)	0.0241 (4)	
C5A	−0.1030 (3)	0.14900 (13)	0.25125 (17)	0.0289 (5)	
C6A	−0.1038 (3)	0.14924 (13)	0.36802 (17)	0.0315 (5)	
C7A	0.0806 (3)	0.18109 (13)	0.42748 (16)	0.0288 (5)	
C8A	0.0996 (4)	0.17678 (15)	0.53295 (16)	0.0389 (5)	
H8AA	−0.0078	0.1535	0.5684	0.047*	
C9A	0.2741 (4)	0.20637 (15)	0.58490 (18)	0.0444 (6)	
H9AA	0.2901	0.2038	0.6567	0.053*	
C10A	0.4267 (4)	0.23999 (16)	0.53074 (17)	0.0432 (6)	
H10B	0.5472	0.2606	0.5674	0.052*	
C11A	0.2440 (3)	0.21495 (12)	0.37991 (15)	0.0246 (4)	
C12A	0.2397 (3)	0.21671 (12)	0.26908 (15)	0.0216 (4)	
C1B	1.0812 (3)	0.39649 (13)	0.95094 (14)	0.0286 (4)	
H1BA	0.9613	0.3742	0.9774	0.034*	
C2B	1.2347 (3)	0.43041 (13)	1.01586 (15)	0.0318 (5)	
H2BA	1.2203	0.4329	1.0866	0.038*	
C3B	1.4085 (3)	0.46050 (13)	0.97591 (15)	0.0305 (5)	
H3BA	1.5180	0.4824	1.0194	0.037*	
C4B	1.4242 (3)	0.45896 (12)	0.87118 (15)	0.0245 (4)	
C5B	1.6077 (3)	0.49192 (13)	0.82586 (16)	0.0284 (5)	
C6B	1.6070 (3)	0.49306 (13)	0.70872 (16)	0.0303 (5)	
C7B	1.4283 (3)	0.45768 (13)	0.65019 (15)	0.0289 (5)	
C8B	1.4144 (4)	0.45842 (15)	0.54424 (17)	0.0393 (6)	
H8BA	1.5239	0.4797	0.5085	0.047*	
C9B	1.2398 (4)	0.42780 (16)	0.49305 (17)	0.0463 (7)	
H9BA	1.2255	0.4283	0.4211	0.056*	
C10B	1.0845 (4)	0.39614 (16)	0.54748 (17)	0.0400 (6)	
H10A	0.9652	0.3746	0.5108	0.048*	
C11B	1.2622 (3)	0.42491 (13)	0.69772 (16)	0.0271 (5)	
C12B	1.2647 (3)	0.42594 (12)	0.80881 (15)	0.0240 (4)	

### Atomic displacement parameters (Å^2^)

**Table:**

	*U*^11^	*U*^22^	*U*^33^	*U*^12^	*U*^13^	*U*^23^
S1	0.0252 (2)	0.0305 (3)	0.0237 (2)	−0.0019 (2)	0.00264 (19)	−0.0019 (2)
S2	0.0248 (2)	0.0314 (3)	0.0226 (2)	−0.0042 (2)	0.00331 (18)	−0.0024 (2)
F11	0.1143 (14)	0.0318 (9)	0.0735 (11)	0.0060 (8)	0.0075 (10)	0.0159 (8)
F12	0.0368 (8)	0.0750 (12)	0.1081 (14)	0.0217 (7)	0.0024 (9)	−0.0140 (11)
F13	0.0798 (11)	0.0579 (11)	0.0435 (8)	0.0118 (8)	0.0049 (8)	−0.0213 (8)
F21	0.0776 (10)	0.0674 (11)	0.0500 (9)	0.0049 (8)	0.0100 (8)	−0.0305 (8)
F22	0.0961 (13)	0.0334 (9)	0.1013 (14)	0.0024 (8)	0.0285 (10)	0.0186 (9)
F23	0.0363 (8)	0.0633 (11)	0.0922 (12)	0.0136 (7)	0.0116 (8)	0.0001 (9)
O11	0.0484 (9)	0.0540 (10)	0.0229 (7)	−0.0107 (8)	−0.0002 (6)	−0.0049 (8)
O12	0.0317 (8)	0.0321 (8)	0.0381 (9)	−0.0046 (6)	0.0024 (6)	0.0069 (7)
O13	0.0217 (7)	0.0516 (11)	0.0399 (8)	−0.0076 (6)	0.0049 (6)	−0.0031 (7)
O21	0.0282 (8)	0.0530 (11)	0.0364 (8)	−0.0054 (7)	0.0052 (6)	−0.0039 (8)
O22	0.0327 (8)	0.0309 (8)	0.0404 (9)	−0.0049 (6)	0.0029 (7)	0.0057 (7)
O23	0.0457 (9)	0.0574 (11)	0.0230 (7)	−0.0121 (8)	0.0003 (6)	−0.0062 (8)
O1A	0.0307 (9)	0.0350 (9)	0.0533 (10)	−0.0088 (7)	−0.0043 (8)	−0.0005 (8)
O2A	0.0434 (9)	0.0411 (10)	0.0555 (10)	−0.0126 (7)	0.0222 (8)	−0.0002 (8)
O1B	0.0320 (8)	0.0346 (9)	0.0485 (9)	−0.0093 (7)	−0.0009 (7)	0.0006 (8)
O2B	0.0392 (9)	0.0411 (10)	0.0517 (10)	−0.0075 (7)	0.0203 (8)	0.0029 (8)
N1A	0.0210 (8)	0.0285 (10)	0.0262 (9)	−0.0027 (7)	−0.0010 (7)	−0.0005 (8)
N2A	0.0312 (9)	0.0345 (11)	0.0293 (9)	0.0017 (8)	−0.0032 (7)	−0.0019 (8)
N1B	0.0229 (8)	0.0292 (10)	0.0253 (8)	−0.0002 (7)	0.0029 (7)	0.0010 (8)
N2B	0.0330 (9)	0.0372 (11)	0.0271 (9)	0.0027 (8)	−0.0025 (7)	−0.0025 (9)
C1	0.0394 (13)	0.0381 (15)	0.0387 (13)	0.0038 (10)	0.0031 (10)	−0.0039 (11)
C2	0.0443 (14)	0.0356 (14)	0.0419 (14)	0.0010 (10)	0.0107 (11)	0.0003 (11)
C1A	0.0306 (11)	0.0344 (13)	0.0300 (11)	−0.0026 (9)	0.0079 (9)	0.0066 (11)
C2A	0.0375 (13)	0.0355 (13)	0.0238 (11)	0.0049 (9)	0.0041 (9)	0.0040 (10)
C3A	0.0310 (11)	0.0265 (12)	0.0319 (12)	0.0017 (8)	−0.0045 (10)	0.0019 (9)
C4A	0.0271 (11)	0.0184 (10)	0.0266 (10)	0.0017 (8)	0.0019 (9)	0.0005 (8)
C5A	0.0258 (11)	0.0200 (11)	0.0417 (13)	0.0024 (8)	0.0073 (9)	0.0004 (10)
C6A	0.0356 (12)	0.0199 (11)	0.0403 (12)	0.0027 (9)	0.0101 (9)	−0.0012 (9)
C7A	0.0334 (11)	0.0234 (11)	0.0302 (11)	0.0021 (8)	0.0058 (9)	−0.0007 (9)
C8A	0.0489 (14)	0.0395 (13)	0.0301 (12)	0.0058 (11)	0.0155 (10)	0.0058 (10)
C9A	0.0619 (17)	0.0460 (16)	0.0253 (11)	0.0075 (12)	0.0035 (11)	0.0035 (11)
C10A	0.0506 (14)	0.0438 (16)	0.0331 (12)	0.0044 (11)	−0.0100 (11)	−0.0022 (11)
C11A	0.0258 (10)	0.0240 (11)	0.0240 (10)	0.0009 (8)	0.0027 (8)	−0.0020 (9)
C12A	0.0198 (10)	0.0210 (11)	0.0247 (10)	0.0019 (7)	0.0063 (8)	0.0006 (9)
C1B	0.0270 (10)	0.0299 (12)	0.0297 (10)	0.0019 (8)	0.0070 (8)	0.0036 (10)
C2B	0.0400 (12)	0.0339 (12)	0.0215 (10)	−0.0014 (9)	0.0024 (9)	0.0033 (9)
C3B	0.0341 (11)	0.0292 (12)	0.0271 (11)	−0.0014 (8)	−0.0056 (9)	−0.0014 (9)
C4B	0.0253 (10)	0.0209 (10)	0.0272 (11)	0.0042 (8)	0.0009 (9)	0.0025 (9)
C5B	0.0277 (11)	0.0211 (11)	0.0361 (11)	0.0032 (8)	0.0011 (9)	0.0037 (9)
C6B	0.0322 (12)	0.0242 (12)	0.0357 (12)	0.0060 (9)	0.0106 (9)	0.0037 (10)
C7B	0.0350 (12)	0.0272 (11)	0.0253 (10)	0.0061 (8)	0.0078 (9)	0.0045 (9)
C8B	0.0535 (15)	0.0378 (13)	0.0282 (12)	0.0080 (11)	0.0137 (11)	0.0028 (11)
C9B	0.0656 (18)	0.0520 (16)	0.0208 (11)	0.0117 (13)	0.0006 (12)	−0.0013 (11)
C10B	0.0466 (13)	0.0407 (15)	0.0312 (12)	0.0047 (11)	−0.0072 (10)	−0.0047 (11)
C11B	0.0315 (11)	0.0258 (11)	0.0243 (10)	0.0066 (9)	0.0033 (9)	0.0019 (9)
C12B	0.0241 (10)	0.0209 (11)	0.0270 (10)	0.0053 (8)	0.0008 (8)	0.0033 (9)

### Geometric parameters (Å, °)

**Table:**

S1—O13	1.4330 (14)	C3A—C4A	1.397 (3)
S1—O11	1.4355 (15)	C3A—H3AA	0.9500
S1—O12	1.4507 (16)	C4A—C12A	1.387 (3)
S1—C1	1.817 (2)	C4A—C5A	1.482 (3)
S2—O21	1.4337 (14)	C5A—C6A	1.547 (3)
S2—O23	1.4362 (14)	C6A—C7A	1.477 (3)
S2—O22	1.4509 (16)	C7A—C11A	1.390 (3)
S2—C2	1.819 (2)	C7A—C8A	1.394 (3)
F11—C1	1.318 (3)	C8A—C9A	1.369 (3)
F12—C1	1.327 (3)	C8A—H8AA	0.9500
F13—C1	1.329 (3)	C9A—C10A	1.381 (4)
F21—C2	1.328 (3)	C9A—H9AA	0.9500
F22—C2	1.328 (3)	C10A—H10B	0.9500
F23—C2	1.329 (3)	C11A—C12A	1.466 (3)
O1A—C5A	1.208 (2)	C1B—C2B	1.382 (3)
O2A—C6A	1.209 (2)	C1B—H1BA	0.9500
O1B—C5B	1.214 (2)	C2B—C3B	1.374 (3)
O2B—C6B	1.216 (2)	C2B—H2BA	0.9500
N1A—C1A	1.341 (3)	C3B—C4B	1.399 (3)
N1A—C12A	1.343 (2)	C3B—H3BA	0.9500
N1A—H1AB	0.8800	C4B—C12B	1.384 (3)
N2A—C11A	1.339 (2)	C4B—C5B	1.476 (3)
N2A—C10A	1.340 (3)	C5B—C6B	1.551 (3)
N1B—C1B	1.338 (2)	C6B—C7B	1.464 (3)
N1B—C12B	1.343 (3)	C7B—C11B	1.396 (3)
N1B—H1BB	0.8800	C7B—C8B	1.399 (3)
N2B—C10B	1.332 (3)	C8B—C9B	1.371 (3)
N2B—C11B	1.338 (3)	C8B—H8BA	0.9500
C1A—C2A	1.373 (3)	C9B—C10B	1.384 (3)
C1A—H1AA	0.9500	C9B—H9BA	0.9500
C2A—C3A	1.380 (3)	C10B—H10A	0.9500
C2A—H2AA	0.9500	C11B—C12B	1.470 (3)
			
O13—S1—O11	115.34 (9)	C8A—C7A—C6A	121.5 (2)
O13—S1—O12	114.30 (9)	C9A—C8A—C7A	119.5 (2)
O11—S1—O12	114.19 (9)	C9A—C8A—H8AA	120.3
O13—S1—C1	105.30 (10)	C7A—C8A—H8AA	120.3
O11—S1—C1	102.40 (10)	C8A—C9A—C10A	118.6 (2)
O12—S1—C1	103.23 (10)	C8A—C9A—H9AA	120.7
O21—S2—O23	115.69 (9)	C10A—C9A—H9AA	120.7
O21—S2—O22	114.19 (9)	N2A—C10A—C9A	123.8 (2)
O23—S2—O22	114.28 (9)	N2A—C10A—H10B	118.1
O21—S2—C2	105.11 (11)	C9A—C10A—H10B	118.1
O23—S2—C2	102.77 (11)	N2A—C11A—C7A	123.88 (19)
O22—S2—C2	102.61 (10)	N2A—C11A—C12A	115.77 (18)
C1A—N1A—C12A	123.07 (17)	C7A—C11A—C12A	120.33 (17)
C1A—N1A—H1AB	118.5	N1A—C12A—C4A	118.63 (17)
C12A—N1A—H1AB	118.5	N1A—C12A—C11A	118.10 (16)
C11A—N2A—C10A	116.7 (2)	C4A—C12A—C11A	123.25 (17)
C1B—N1B—C12B	122.78 (17)	N1B—C1B—C2B	120.40 (18)
C1B—N1B—H1BB	118.6	N1B—C1B—H1BA	119.8
C12B—N1B—H1BB	118.6	C2B—C1B—H1BA	119.8
C10B—N2B—C11B	116.6 (2)	C3B—C2B—C1B	118.62 (19)
F11—C1—F12	108.5 (2)	C3B—C2B—H2BA	120.7
F11—C1—F13	107.22 (19)	C1B—C2B—H2BA	120.7
F12—C1—F13	107.7 (2)	C2B—C3B—C4B	119.98 (19)
F11—C1—S1	111.27 (16)	C2B—C3B—H3BA	120.0
F12—C1—S1	110.05 (16)	C4B—C3B—H3BA	120.0
F13—C1—S1	111.92 (16)	C12B—C4B—C3B	119.40 (19)
F21—C2—F22	108.0 (2)	C12B—C4B—C5B	119.35 (18)
F21—C2—F23	107.5 (2)	C3B—C4B—C5B	121.25 (18)
F22—C2—F23	107.18 (19)	O1B—C5B—C4B	122.31 (19)
F21—C2—S2	111.81 (17)	O1B—C5B—C6B	119.62 (18)
F22—C2—S2	111.26 (16)	C4B—C5B—C6B	118.06 (17)
F23—C2—S2	110.84 (16)	O2B—C6B—C7B	124.3 (2)
N1A—C1A—C2A	119.98 (19)	O2B—C6B—C5B	117.92 (19)
N1A—C1A—H1AA	120.0	C7B—C6B—C5B	117.79 (18)
C2A—C1A—H1AA	120.0	C11B—C7B—C8B	117.6 (2)
C1A—C2A—C3A	119.2 (2)	C11B—C7B—C6B	121.40 (19)
C1A—C2A—H2AA	120.4	C8B—C7B—C6B	121.0 (2)
C3A—C2A—H2AA	120.4	C9B—C8B—C7B	118.7 (2)
C2A—C3A—C4A	119.6 (2)	C9B—C8B—H8BA	120.7
C2A—C3A—H3AA	120.2	C7B—C8B—H8BA	120.7
C4A—C3A—H3AA	120.2	C8B—C9B—C10B	119.2 (2)
C12A—C4A—C3A	119.47 (18)	C8B—C9B—H9BA	120.4
C12A—C4A—C5A	119.66 (18)	C10B—C9B—H9BA	120.4
C3A—C4A—C5A	120.85 (19)	N2B—C10B—C9B	123.9 (2)
O1A—C5A—C4A	122.9 (2)	N2B—C10B—H10A	118.0
O1A—C5A—C6A	119.47 (19)	C9B—C10B—H10A	118.0
C4A—C5A—C6A	117.65 (18)	N2B—C11B—C7B	124.1 (2)
O2A—C6A—C7A	123.4 (2)	N2B—C11B—C12B	116.08 (19)
O2A—C6A—C5A	118.55 (19)	C7B—C11B—C12B	119.79 (18)
C7A—C6A—C5A	118.00 (18)	N1B—C12B—C4B	118.77 (18)
C11A—C7A—C8A	117.5 (2)	N1B—C12B—C11B	117.69 (17)
C11A—C7A—C6A	120.98 (19)	C4B—C12B—C11B	123.52 (18)
			
O13—S1—C1—F11	−63.50 (18)	C1A—N1A—C12A—C11A	177.25 (18)
O11—S1—C1—F11	57.46 (18)	C3A—C4A—C12A—N1A	0.5 (3)
O12—S1—C1—F11	176.32 (16)	C5A—C4A—C12A—N1A	179.33 (19)
O13—S1—C1—F12	176.18 (16)	C3A—C4A—C12A—C11A	−177.69 (18)
O11—S1—C1—F12	−62.86 (19)	C5A—C4A—C12A—C11A	1.1 (3)
O12—S1—C1—F12	56.00 (19)	N2A—C11A—C12A—N1A	−0.5 (3)
O13—S1—C1—F13	56.44 (19)	C7A—C11A—C12A—N1A	−178.6 (2)
O11—S1—C1—F13	177.40 (16)	N2A—C11A—C12A—C4A	177.71 (19)
O12—S1—C1—F13	−63.74 (19)	C7A—C11A—C12A—C4A	−0.4 (3)
O21—S2—C2—F21	−55.78 (19)	C12B—N1B—C1B—C2B	−0.7 (3)
O23—S2—C2—F21	−177.22 (16)	N1B—C1B—C2B—C3B	−1.4 (3)
O22—S2—C2—F21	63.91 (19)	C1B—C2B—C3B—C4B	2.2 (3)
O21—S2—C2—F22	65.10 (18)	C2B—C3B—C4B—C12B	−0.9 (3)
O23—S2—C2—F22	−56.34 (18)	C2B—C3B—C4B—C5B	179.1 (2)
O22—S2—C2—F22	−175.21 (16)	C12B—C4B—C5B—O1B	−177.13 (19)
O21—S2—C2—F23	−175.74 (16)	C3B—C4B—C5B—O1B	2.9 (3)
O23—S2—C2—F23	62.82 (18)	C12B—C4B—C5B—C6B	3.9 (3)
O22—S2—C2—F23	−56.05 (18)	C3B—C4B—C5B—C6B	−176.08 (17)
C12A—N1A—C1A—C2A	0.5 (3)	O1B—C5B—C6B—O2B	−3.0 (3)
N1A—C1A—C2A—C3A	0.5 (3)	C4B—C5B—C6B—O2B	176.0 (2)
C1A—C2A—C3A—C4A	−1.0 (3)	O1B—C5B—C6B—C7B	178.08 (19)
C2A—C3A—C4A—C12A	0.5 (3)	C4B—C5B—C6B—C7B	−2.9 (3)
C2A—C3A—C4A—C5A	−178.3 (2)	O2B—C6B—C7B—C11B	−177.9 (2)
C12A—C4A—C5A—O1A	−179.9 (2)	C5B—C6B—C7B—C11B	0.9 (3)
C3A—C4A—C5A—O1A	−1.0 (3)	O2B—C6B—C7B—C8B	−0.5 (3)
C12A—C4A—C5A—C6A	0.9 (3)	C5B—C6B—C7B—C8B	178.34 (19)
C3A—C4A—C5A—C6A	179.67 (18)	C11B—C7B—C8B—C9B	0.5 (3)
O1A—C5A—C6A—O2A	−2.9 (3)	C6B—C7B—C8B—C9B	−177.1 (2)
C4A—C5A—C6A—O2A	176.4 (2)	C7B—C8B—C9B—C10B	−0.9 (4)
O1A—C5A—C6A—C7A	177.2 (2)	C11B—N2B—C10B—C9B	0.0 (4)
C4A—C5A—C6A—C7A	−3.5 (3)	C8B—C9B—C10B—N2B	0.7 (4)
O2A—C6A—C7A—C11A	−175.5 (2)	C10B—N2B—C11B—C7B	−0.6 (3)
C5A—C6A—C7A—C11A	4.3 (3)	C10B—N2B—C11B—C12B	177.13 (18)
O2A—C6A—C7A—C8A	5.9 (3)	C8B—C7B—C11B—N2B	0.3 (3)
C5A—C6A—C7A—C8A	−174.27 (19)	C6B—C7B—C11B—N2B	177.9 (2)
C11A—C7A—C8A—C9A	0.7 (3)	C8B—C7B—C11B—C12B	−177.29 (18)
C6A—C7A—C8A—C9A	179.4 (2)	C6B—C7B—C11B—C12B	0.3 (3)
C7A—C8A—C9A—C10A	0.2 (4)	C1B—N1B—C12B—C4B	2.1 (3)
C11A—N2A—C10A—C9A	−0.7 (4)	C1B—N1B—C12B—C11B	−176.33 (18)
C8A—C9A—C10A—N2A	−0.2 (4)	C3B—C4B—C12B—N1B	−1.3 (3)
C10A—N2A—C11A—C7A	1.7 (3)	C5B—C4B—C12B—N1B	178.77 (18)
C10A—N2A—C11A—C12A	−176.33 (18)	C3B—C4B—C12B—C11B	177.07 (17)
C8A—C7A—C11A—N2A	−1.8 (3)	C5B—C4B—C12B—C11B	−2.9 (3)
C6A—C7A—C11A—N2A	179.60 (19)	N2B—C11B—C12B—N1B	1.3 (3)
C8A—C7A—C11A—C12A	176.19 (18)	C7B—C11B—C12B—N1B	179.1 (2)
C6A—C7A—C11A—C12A	−2.4 (3)	N2B—C11B—C12B—C4B	−177.06 (19)
C1A—N1A—C12A—C4A	−1.0 (3)	C7B—C11B—C12B—C4B	0.7 (3)

### Hydrogen-bond geometry (Å, °)

**Table:**

*D*—H···*A*	*D*—H	H···*A*	*D*···*A*	*D*—H···*A*
N1A—H1AB···O22	0.88	2.01	2.830 (2)	154
N1B—H1BB···O12	0.88	2.02	2.835 (2)	154
C1A—H1AA···O11^i^	0.95	2.37	3.141 (3)	138
C1A—H1AA···O23	0.95	2.39	3.190 (3)	141
C1B—H1BA···O11	0.95	2.41	3.206 (3)	142
C1B—H1BA···O23^ii^	0.95	2.40	3.175 (2)	139
C2A—H2AA···O13^i^	0.95	2.49	3.395 (3)	159
C9A—H9AA···O13	0.95	2.43	3.320 (3)	156
C2B—H2BA···O21^ii^	0.95	2.49	3.384 (3)	158
C9B—H9BA···O21	0.95	2.43	3.328 (3)	159

Symmetry codes: (i) *x*, *y*, *z*−1; (ii) *x*, *y*, *z*+1.
